# Daytime nap controls toddlers’ nighttime sleep

**DOI:** 10.1038/srep27246

**Published:** 2016-06-09

**Authors:** Machiko Nakagawa, Hidenobu Ohta, Yuko Nagaoki, Rinshu Shimabukuro, Yoko Asaka, Noriko Takahashi, Takayo Nakazawa, Yousuke Kaneshi, Keita Morioka, Yoshihisa Oishi, Yuriko Azami, Mari Ikeuchi, Mari Takahashi, Michio Hirata, Miwa Ozawa, Kazutoshi Cho, Isao Kusakawa, Hitoshi Yoda

**Affiliations:** 1 Department of Developmental Disorders, National Institute of Mental Health, National Center of Neurology and Psychiatry, 4-1-1 Ogawa-higashi-cho, Kodaira, Tokyo 187-8553, Japan; 2 Department of Pediatrics, St. Luke’s International Hospital, 9-1 Akashi-cho, Chuo-ku, Tokyo 104-8560, Japan; 3 Faculty of Health Sciences, Hokkaido University, N12, W5, Kita-ku, Sapporo 060-0812, Japan; 4 Maternity and Perinatal Care Center, Hokkaido University Hospital, N15, W7, Kita-ku, Sapporo 060-8638, Japan; 5 Department of Pediatrics, Japanese Red Cross Medical Center, 4-1-22 Hiroo, Shibuya-ku, Tokyo 150-8935, Japan; 6 Department of Neonatology, Toho University Omori Medical Center, 6-11-1 Omori-nishi, Ota-ku, Tokyo 143-8541, Japan

## Abstract

Previous studies have demonstrated that afternoon naps can have a negative effect on subsequent nighttime sleep in children. These studies have mainly been based on sleep questionnaires completed by parents. To investigate the effect of napping on such aspects of sleep quality, we performed a study in which child activity and sleep levels were recorded using actigraphy. The parents were asked to attach actigraphy units to their child’s waist by an adjustable elastic belt and complete a sleep diary for 7 consecutive days. 50 healthy young toddlers of approximately 1.5 years of age were recruited. There was a significant negative correlation between nap duration and both nighttime sleep duration and sleep onset time, suggesting that long nap sleep induces short nighttime sleep duration and late sleep onset time. We also found a significant negative correlation between nap timing and nighttime sleep duration and also a significant positive correlation between nap timing and sleep onset time, suggesting that naps in the late afternoon also lead to short nighttime sleep duration and late sleep onset. Our findings suggest that duration-controlled naps starting early in the afternoon can induce a longer nighttime sleep in full-term infants of approximately 1.5 years of age.

The first few years of life are associated with marked changes in the amount and distribution of sleep. Between 0 and 5 years of age, the frequency of afternoon naps declines and the biphasic sleep-wake pattern gives way to a consolidated pattern of sleep occurring only at night. Moreover, the basic sleep structure for young children has been reported to be established by 1.5 years of age and remains similar at least until 5 years of age^1^,^2^,^3^,^4^,^5^,^6^.

There have been several population studies concerning changes in the amount and diurnal distribution of infants’ and children’s sleep mainly based on sleep questionnaires completed by parents, indicating that the longer the nap durations of children, the later they went to bed^7^,^8^,^9^,^10^. Using activity recording devices, Acebo *et al*.^1^ also confirmed similar effects of nap duration on the nighttime sleep of children. Although their study greatly contributed to the scientific understanding of sleep development of young children between 1 and 5 years of age, their study population was relatively small. In particular, the number of children aged 1.5 years-a key age at which children form their sleep structure-was less than 30. In addition, they calculated nap duration with a time resolution of 30 minute units, and may have underestimated the effects of nap on bedtime timing since the nap duration of children aged 1.5 years is a relatively short duration of approximately 2 hours^1^. A recent systematic review also indicated that previous studies on infant sleep rely mostly on parent reports and suggested that the studies should use independent physiological measurement of sleep behaviors. The review concluded that there is consistent, but low-quality, evidence that napping is increasingly associated with delayed night sleep onset and disrupted night sleep at least at >2 years of age^6^.

In the current study, to examine the effects of daytime nap on the nighttime sleep quality of young toddlers of approximately 1.5 years of age, we investigated the daytime napping patterns and nocturnal sleep-wake patterns of 50 children using actigraphy and also examined if long nap duration actually deteriorates sleep quality in the subsequent night among toddlers. Our study is the first report on trends in actigraphic findings on daytime activity and nighttime sleep of young toddlers of 1.5 years of age, an age at which a regular child health examination is performed nationwide in Japan.

## Results

**Figure 1:**
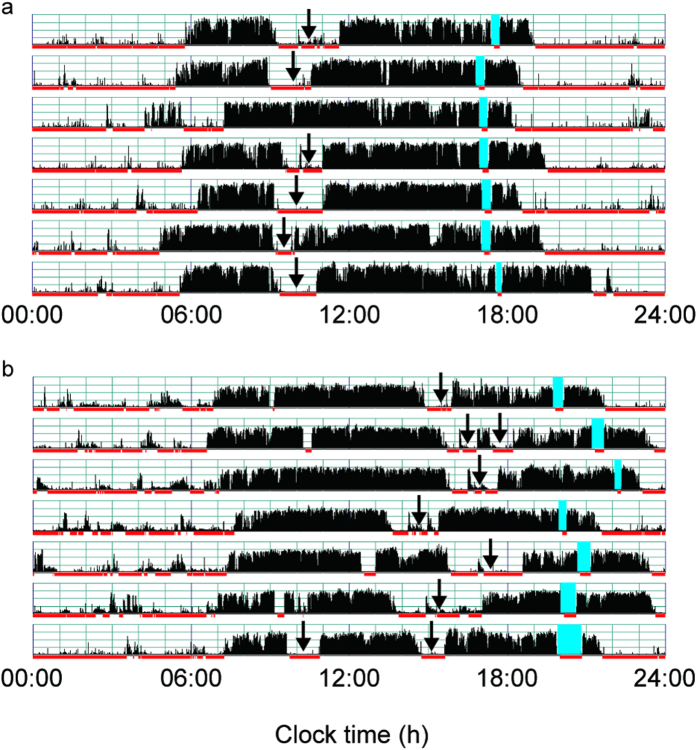
The actograms show representative daily activity-rest patterns of full-term infants of approximately 1.5 years of age with advanced nap phases (**a**) and delayed nap phases (**b**). The vertical axis shows the 7 consecutive observation days and the horizontal axis shows the course of each 24 h day from 00:00 h (12:00 am) to 24:00 h (12:00 am). Activity counts per minute are represented by the height of the vertical black bars on each actogram. The arrows and the blue rectangles indicate naps and bathing periods, respectively. The red underlines are the periods that were automatically judged as sleep periods by the actigraph software.

**Figure 2:**
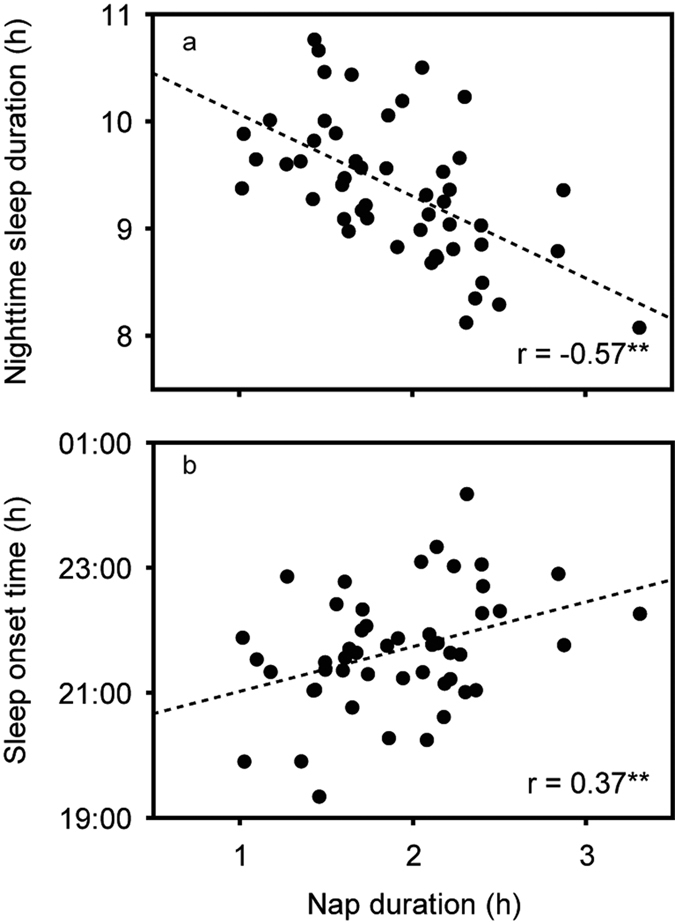
Correlations of nap duration with nighttime sleep duration (**a**) and sleep onset time (**b**) in full-term infants (**p < 0.01).

**Figure 3:**
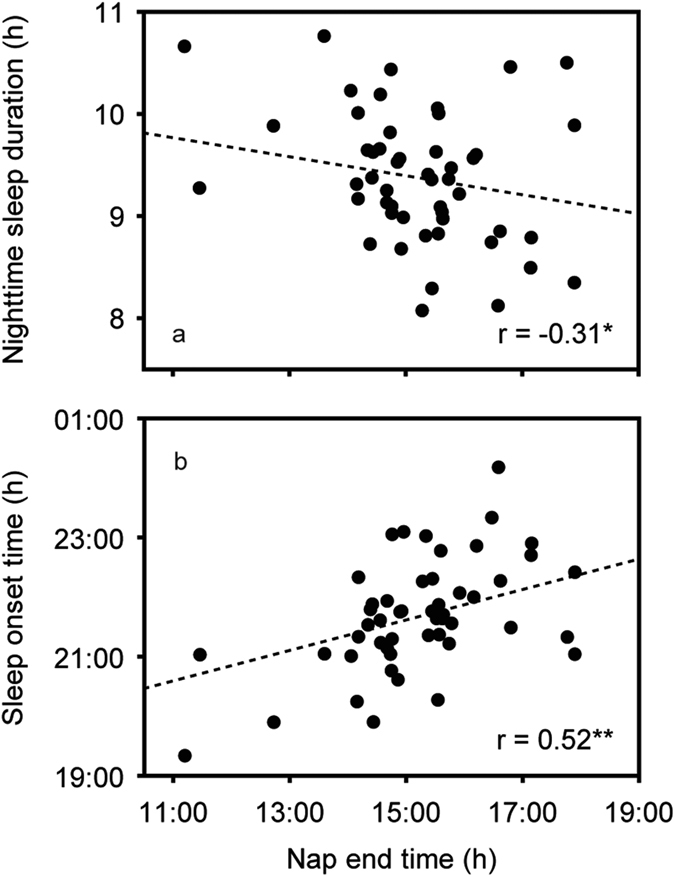
Correlations of nap-end time with nighttime sleep duration (**a**) and sleep onset time (**b**) in full-term infants (*p < 0.05, **p < 0.01).

The characteristics of the 50 infants are shown in Table 1. Toddlers’ sleep environment is shown in Table 2. Toddlers’ sleep variables such as average bedtime, average wake time, average nighttime sleep duration, and average nap duration are also shown in Table 3. No differences were found between boys and girls in any of the sleep variables (t-test, p > 0.05). In addition, no differences were found in the durations of naps and nighttime sleep between weekdays and weekends (t-test, p > 0.05). Figure 1 demonstrates representative daily activity-rest patterns of full-term infants of approximately 1.5 years of age, indicating the existence of various nap patterns among toddlers. There were significant negative correlations between nap duration and nighttime sleep duration (r = −0.57, p = 0.000), and also positive correlations between nap duration and sleep onset time (r = 0.37, p = 0.008) and nap-end time (r = 0.36, p = 0.011) (Table 4, Fig. 2). Like nap duration, we also found significant negative correlations between nap-end time and nighttime sleep duration (r = −0.31, p = 0.028), and positive correlation with sleep onset time (r = 0.52, p = 0.000) and nap duration (r = 0.36, p = 0.011) (Table 4, Fig. 3). The data suggested that both long nap duration and naps in the late afternoon induce short nighttime sleep duration and late sleep onset. The analysis also suggested that late nap-end time leads to long nap duration since a significant positive correlation between the nap-end time and nap duration were found.

## Discussion

The present study indicates two significant factors influencing the nighttime sleep of toddlers at approximately 1.5 years of age. First, our actigraphic data confirm that, in 1.5-year-old children, naps affect nighttime sleep, consistent with previous studies based on sleep questionnaires, which have indicated that the longer the nap duration of children, the later they went to bed. Using actigraphy, the current study also found that ‘longer nap duration’ is significantly associated with shorter nighttime sleep duration and later sleep onset. However, our data differs from the actigraphic data of 1.5-year-old children from Acebo *et al*.^1^, in that their data did not show a significant association between nap duration and sleep onset time. The difference may come mainly from the time resolution used for nap recording (30 minute units and 1 minute units for Acebo *et al*.^1^ and the present study, respectively) because the toddlers’ average nap duration of 1.9 ± 0.8 hours in the present study could be under- or over-evaluated by a parents’ sleep diary using a 30-minute time resolution, which would tend to cause larger data variation. In addition, difference between the data by Acebo *et al*.^1^ and our data may also come from the difference in the sample number of children for statistical analysis (29 and 50 children for Acebo *et al*.^1^ and the present study, respectively). In addition a previous questionnaire study^11^ reporting that children (50–72 months, n = 168) who experienced >60 minutes of mandatory nap time in child care had significantly less nighttime sleep than those with 0 to 60 minutes of mandatory nap time. The same group also closely investigated the nap patterns of preschool children (3–5 years, n = 2,114)^12^. They reported that 83.5% of child care settings implemented a mandatory nap time (range = 15–145 min) while 14.2% provided alternate activities for children throughout the nap time period and also found that 31% of all children napped during nap times.

Second, the current study also described a new finding, which was that, like nap duration, “later nap-ending time” is significantly associated with shorter nighttime sleep duration, later sleep onset time, and also nap durations. This suggests that parents might be able to prolong toddlers’ nighttime sleep duration simply by advancing the nap-ending time since correlation analysis indicates that nap durations might be significantly affected by nap-ending time. In addition, interestingly, both nap durations and nap-ending time do not have significant correlations with total sleep duration, which is the summation of nap and nighttime sleep durations. The data suggest that nap duration and nap-ending time simply controls the distribution ratio between nap and nighttime sleep durations in total sleep duration but does not affect the amount of total sleep duration. There is still an ongoing debate whether either nap or nighttime sleep is more valuable to achieve proper child health and development or if only total sleep duration is an important factor for healthy child development. However, a recent study at least reported that daytime naps improve word learning in toddlers of 16 months of age^13^,^14^. A further study using additional physiological parameters will be required to obtain appropriate answers regarding sleep issues in child development.

There are a number of limitations that should be considered in the interpretation of the results. First, because of the relatively small sample size (n = 50), this study did not fully perform analysis on sex difference in actigraph variables. Since the study population is also not well-balanced between boys (n = 32) and girls (n = 18), a study population with a larger sample size will be required to make a satisfactory statistical analysis on the effect of sex difference on toddlers’ sleep variables in any future study. In accordance with Acebo’s study, however, the present study confirms that effect of sex difference was not significant for any actigraph variable among the toddlers at approximately 1.5 years of age (Table 3). Second, because a detailed parent questionnaire was not included in the study, we were not able to precisely examine the similarities and dissimilarities between actigraph data and parent questionnaires. In a previous study, Acebo *et al*.^1^ found well-matched data between actigraph data and parent-reported observations. Finally, the present study did not fully investigate the effect of nap duration or timing on night time sleep because we did not perform an RCT study, which would expose children to two different nap durations. Such an RCT study would further strengthen the findings of the present study that daily naps with short periods and starting early in the afternoon can induce a longer nighttime sleep for toddlers at approximately 1.5 years of age.

## Methods

### Participants

Young toddlers of approximately 1.5 years of age were recruited at the Children’s Clinic of St. Luke’s International Hospital (Tokyo, Japan). Inclusion criteria were as follows: (1) full-term pregnancy (defined as ≥37 weeks’ gestational age) and (2) the absence of known physical or mental disability or of severe developmental delay in the infant. Exclusion criteria was parental language difficulties. Of 72 eligible toddlers, 22 were excluded because sleep data were invalid due to technical problems with the activity recording devices or incomplete description of sleep diary. The final sample thus consisted of 50 young toddlers (32 boys, 18 girls). The ethics committees of the St. Luke’s International Hospital and the National Center of Neurology and Psychiatry approved the study protocol (UMIN000019696) and all procedures were carried out in accordance with the approved guidelines. Written informed consent was obtained from the parents.

### Activity and sleep assessment

#### Actigraphy

For activity and sleep measurement we used actigraphy. Actigraphy is based on a miniature wristwatch-like accelerometer which is attached to the wrist, ankle or waist and continuously records movement for an extended period. The actigraphy device used in the present study was the Actigraph (Micro-mini RC, Ambulatory Monitoring Inc., NY, USA). The parents were asked to attach the Actigraphs to their child’s waist with an adjustable elastic belt for 7 consecutive days. Waist attachment was chosen as we found it less disturbing than wrist or ankle attachment and actigraphic data has been reported to be reliable at various attachment locations, including the wrist, ankles, and waist. Previous studies have also demonstrated that a minimum of 7 nights was necessary to obtain reliable data^15^.

Motility levels were sampled in the zero-crossing mode in 1-min epochs. The resolution of the Actigraph was set at 0.01 G/s. The activity data recorded by the Actigraph was later downloaded using ActMe software (ver. 3.10.0.3,Ambulatory Monitoring Inc.), and then sleep measurements were analyzed using Action-W software (ver. 2.4.20, Ambulatory Monitoring Inc.). During the study, time intervals when the device was removed, for example, during bathing, were recorded in a sleep diary by parents.

#### Sleep diary

Parents were instructed to complete a sleep diary for the 7-day period while their child was wearing the Actigraph. The diary consisted of seven 24-hr single-sheet schedules, in which parents were asked to write information such as time of nap, going in/out of bed, bathing and night awakenings of which they were aware.

### Statistical analysis

Statistical analyses were performed with SPSS Statistics 21.0 (IBM Corp. Armonk, NY, USA). Summary measurements are presented as means ± s.d.s. Pearson correlation was used to assess associations between variables, respectively. The gender difference in sleep environment and bedtime routine was analyzed using a χ^2^ test for categorical data and a t-test for continuous data.

## Additional Information

**How to cite this article**: Nakagawa, M. *et al*. Daytime nap controls toddlers’ nighttime sleep. *Sci. Rep*. **6**, 27246; doi: 10.1038/srep27246 (2016).

## Figures and Tables

**Table 1:** Characteristics of participants.

Gestational age at birth (weeks), mean ± s.d.	39.5 ± 1.2
Birth weight (g), mean ± s.d.	3092.5 ± 364.7
No. of toddlers (boys: girls)	50 (32:18)
Maternal age at birth, mean ± s.d.	35.3 ± 3.9
Birth order	
First born	35
Subsequent born	15
Months of age at actigraph recording, mean ± s.d.	19.1 ± 1.2

**Table 2:** Sleep Environment and Bedtime Routine (number (%) or mean ± s.d.).

		Boys(n = 32)	Girls(n = 18)	p-value	
Home environment					
Siblings	Yes	10(31.3)	5(27.8)		
	No	22(68.8)	13(72.2)	0.797	
Child has own room	Yes	28(87.5)	17(94.4)		
	No	4(12.5)	1(5.55)	0.432	
Falls asleep on parent’s bed	Yes	20(62.5)	11(61.1)		
	No	12(37.5)	7(38.9)	0.923	
Parent has concerns about child’s sleep					
	Yes	16(50)	8(44.4)		
	No	16(50)	10(55.5)	0.706	
Nap during weeks					
	Yes	32(100)	18(100)		
	No	0(0)	0(0)		
Parent present when child is falling asleep					
	Yes	28(87.5)	14(77.7)		
	No	4(12.5)	4(22.2)	0.368	
Bed time					
Weekday		21:04 ± 56	21:20 ± 50	0.161	
Weekend		21:09 ± 60	21:33 ± 60	0.086	
Wake time					
Weekday		7:02 ± 49	7:10 ± 38	0.272	
Weekend		7:05 ± 58	7:16 ± 50	0.247	

**Table 3:** Sleep Variables (mean ± s.d.).

Sleep Variables	Total (n = 50)	Boys (n = 32)	Girls (n = 18)
Bedtime	21:12 ± 1:04	21:06 ± 0:55	21:24 ± 0:51
Sleep onset time	21:40 ± 1:07	21:35 ± 1:00	21:49 ± 0:51
Wake time	7:05 ± 0:56	7:02 ± 0:49	7:11 ± 0:40
Sleep latency	27.3 ± 21.3	28.5 ± 13.6	25.3 ± 10.8
Nighttime sleep duration	9.4 ± 1.0	9.4 ± 0.7	9.3 ± 0.6
Nap duration	1.9 ± 0.8	1.9 ± 0.5	1.9 ± 0.5
Total sleep duration	11.3 ± 1.0	11.3 ± 0.6	11.2 ± 0.6
Nap end time	15:13 ± 2:08	15:22 ± 1:18	14:55 ± 1:26
Sleep efficiency	87.6 ± 9.3	87.4 ± 7.3	88.2 ± 8.0
WASO (wake after sleep onset)	68.3 ± 51.6	70.1 ± 41.4	63.7 ± 42.3

**Table 4:** Correlations between sleep variables (*p < 0.05, **p < 0.01).

Sleep variables	Nap duration		Nap end time	
	r	p	r	p
Bedtime	0.38	0.006**	0.52	0.000**
Sleep onset time	0.37	0.008**	0.52	0.000**
Wake time	−0.06	0.662	0.37	0.007**
Sleep latency	0.02	0.905	0.08	0.576
Nighttime sleep duration	−0.57	0.000**	−0.31	0.028*
Nap duration	–	–	0.36	0.011*
Total sleep duration	0.21	0.141	−0.051	0.727
Nap end time	0.36	0.011*	–	–
Sleep efficiency	0.06	0.661	−0.15	0.284
WASO (wake after sleep outset)	−0.15	0.314	0.11	0.453
